# Decoding the biosynthesis and function of diphthamide, an enigmatic modification of translation elongation factor 2 (EF2)

**DOI:** 10.15698/mic2014.06.151

**Published:** 2014-05-20

**Authors:** Raffael Schaffrath, Michael J. R. Stark

**Affiliations:** 1 Institut für Biologie, Abteilung Mikrobiologie, Universität Kassel, D-34132 Kassel, Germany.; 2 Centre for Gene Regulation & Expression, College of Life Sciences, MSI/WTB Complex, University of Dundee, Dundee DD1 5EH, Scotland, UK.

**Keywords:** EF2 modification, diphthamide, DPH1-DPH7, diphtheria toxin, sordarin fungicides, budding yeast, Saccharomyces cerevisiae

## Abstract

Diphthamide is a highly conserved modification of archaeal and eukaryal translation elongation factor 2 (EF2) and yet why cells need EF2 to contain diphthamide is unclear. In yeast, the first steps of diphthamide synthesis and the genes (*DPH1-DPH5*) required to form the intermediate diphthine are well-documented. However, the last step, amidation of diphthine to diphthamide, had largely been ill-defined. Remarkably, through mining genome-wide synthetic gene array (SGA) and chemical genomics databases, recent studies by Uthman *et al.* [PLoS Genetics (2013) 9, e1003334] and Su *et al.* [Proc. Natl. Acad. Sci. USA (2012) 109, 19983-19987] have identified two more diphthamide players, *DPH6 *and *DPH7*. Consistent with roles in the amidation step, *dph6 *and *dph7* deletion strains fail to complete diphthamide synthesis and accumulate diphthine-modified EF2. In contrast to Dph6, the catalytically relevant amidase, Dph7 appears to be regulatory. As shown by Uthman *et al.*, it promotes dissociation of diphthine synthase (Dph5) from EF2, allowing diphthine amidation by Dph6 to occur and thereby coupling diphthine synthesis to the terminal step in the pathway. Remarkably, the study by Uthman *et al*. suggests that Dph5 has a novel role as an EF2 inhibitor that affects cell growth when diphthamide synthesis is blocked or incomplete and, importantly, shows that diphthamide promotes the accuracy of EF2 performance during translation.

 Diphthamide is a posttranslationally modified histidine residue in EF2 whose formation is conserved among archaea and eukarya. The name diphthamide refers to its target function for corynebacterial diphtheria toxin (DT), the pathogenic agent causative of the human disease diphtheria. Strikingly, DT hijacks diphthamide on EF2 to inactivate the translation factor by ADP ribosylation. In addition, diphthamide promotes growth inhibition by sordarin, a fungicide inactivating EF2 via stalling the modified translation factor on ribosomes. Taken together, EF2 constitutes an ‘Achilles heel’, study of which has provided important insight into the pathobiological relevance of diphthamide for eukaryotes. Previous screens for isolating *Saccharomyces cerevisiae* mutants resistant against DT and sordarin had identified genes (*DPH1-DPH5*) with roles in the first two diphthamide pathway steps required for making the intermediate diphthine (Figure 1A). The study in yeast by Uthman *et al.* [PLoS Genetics (2013) 9, e1003334] aimed at characterizing the last and ill-defined step of the diphthamide pathway, conversion of diphthine to diphthamide (Figure 1A).

**Figure 1:**
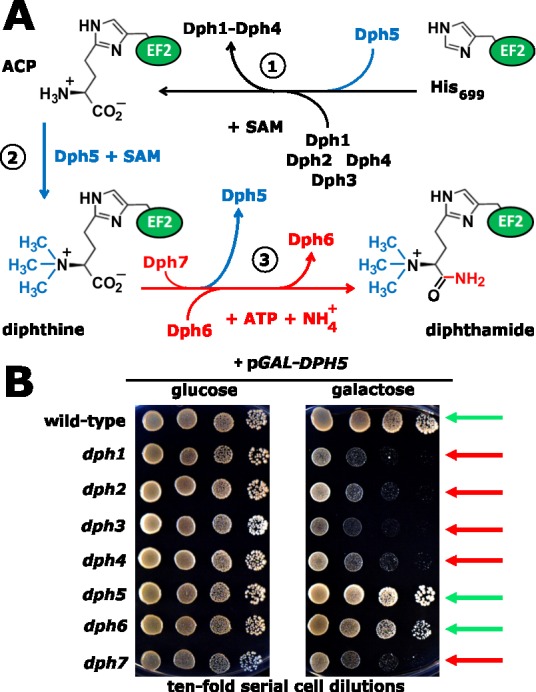
FIGURE 1: The diphthamide synthesis pathway of budding yeast and growth-related traits of diphthamide mutants in response to excess levels of diphthine synthase Dph5. The illustration incorporates evidence from the study of Uthman *et al.* [PLoS Genetics (2013) 9, e1003334] on novel roles for Dph6 and Dph7 in diphthine amidation, Dph7-dependent dissociation of Dph5 from EF2 and *DPH5 *overexpression toxicity. **(A)** The diphthamide pathway. For early roles played by Dph1-Dph5 in the pathway steps 1 & 2, see text. The formerly ill-defined step 3, conversion of diphthine to diphthamide by amidation, is highlighted (red label). It involves ATP and ammonium cofactors in a reaction catalysed by Dph6 and regulated by Dph7. The latter is involved in displacement of Dph5 from EF2 prior to amidation thus coupling steps 2 & 3 of the pathway. **(B)** *DPH5 *overexpression toxicity. Galactose-inducible *DPH5 *overexpression (p*GAL-DPH5*) (right panel) hardly affects wild-type, *dph5* or *dph6* cell growth (green arrows) but is inhibitory to the growth of step 1 (*dph1-dph4*) and step 3 (*dph7*) pathway mutants (red arrows). Glucose repression (left panel) served as a growth control reference. Abbreviations: ACP, 2-[3-amino-carboxyl-propyl]-histidine; SAM, *S*-adenosyl-methionine.

In yeast, the first step in diphthamide formation involves transfer to the imidazole ring of histidine H_699_ in EF2 of a 3-amino-3-carboxypropyl (ACP) radical from *S*-adenosylmethionine (SAM). This generates the ACP intermediate and requires Dph1-Dph4, where the iron-sulfur cluster proteins Dph1/Dph2 are directly involved in ACP radical generation and interact with Dph3, which serves as electron carrier to maintain their redox state (Figure 1A). Dph3 (originally identified as Kti11) also partners with Elongator, an acetylase complex essential for a tRNA modification pathway that may involve radical SAM chemistry as well. Synthesis of diphthine, the second intermediate (Figure 1A) also requires SAM, but as methyl donor for ACP methylation by diphthine synthase (Dph5). Finally, amidation converts diphthine to diphthamide in an ATP-dependent step, which at the onset of the work reported by Uthman *et al.* had been elusive (Figure 1A). Once fully modified by diphthamide, EF2 can be inhibited by sordarin or DT. Interestingly, no DT resistant mutants defective in the amidation step had ever been isolated. This is probably because diphthine is also weakly ADP ribosylated by DT, such that amidase mutants display some DT sensitivity *in vivo* and thus escaped identification in DT resistance screens. However, an indication that additional factors were needed for diphthamide synthesis in both yeast (*YLR143w*; *YBR246w*) and mammals (WDR85) had come from independent work by Thijn Brummelkamp (The Netherlands Cancer Institute), Hening Lin (Cornell University, USA) and from the preliminary studies of Uthman *et al.* By exploiting yeast genome-wide SGA (DRYGIN) and chemical genomics (FitDB) databases, further mining of the *DPH1-DPH5* genetic interaction landscape revealed that *YLR143W *(*DPH6*) and *YBR246W *(*DPH7*) cluster tightly within the *DPH1-DPH5* network and their robust correlations predicted novel roles within the diphthamide pathway. Consistently, the study by Uthman *et al.* validates these predictions with genetic, biochemical and molecular methodologies showing *DPH6 *and *DPH7* indeed operate in the terminal amidation step of the diphthamide pathway (Figure 1A). Thus mass spectrometry demonstrates that *dph6 *and *dph7* deletion strains specifically accumulate the diphthine-modified form of EF2, and their failure to complete diphthine amidation results in loss of ADP ribosylation acceptor activity of EF2 in the presence of DT *in vitro*. Moreover, the amidation defect partially protects against DT *in vivo* and correlates with resistance to EF2 inactivation and growth inhibition by sordarin, collectively traits typical of *bona fide* diphthamide synthesis mutants.

As for the amidation step, a parallel report by the group of Hening Lin [Proc. Natl. Acad. Sci. USA (2012) 109, 19983-19987] identified Dph6 as an ATP-dependent diphthamide synthetase that catalyses the reaction using ammonium as a cofactor (Figure 1A). What then is the role of Dph7, which is also needed for diphthine amidation? Its domain structure, with well-defined WD40 repeats, suggested it might mediate protein-protein interactions as an adaptor for diphthine amidation. However, this is at odds with failure by Uthman *et al.* to detect interaction between Dph7 and either Dph6 or EF2. Conversely, Uthman *et al.* show that Dph7 is required for proper dissociation of Dph5 from EF2, and incomplete diphthamide synthesis in the absence of Dph7 drastically increases the pools of EF2 bound to Dph5. This indicates that their association is kept in check by Dph7, a notion supported by similar findings for WDR85, the mammalian homolog of Dph7. Thus Dph7 appears to act as a license factor that couples the second step, diphthine synthesis by Dph5, to the final step of diphthine amidation by Dph6 (Figure 1A). However, while clearly required for diphthamide formation, Dph7 has other potential roles in RNA polymerase I regulation (Rrt2) and retromer mediated endosomal recycling (Ere1). Given the currently cryptic connection of both these seemingly unrelated functions to diphthamide synthesis and EF2, a full understanding of the role of Dph7 requires further investigation.

Strikingly, Uthman *et al. *report that unmodified EF2 also shows strongly enhanced binding to Dph5. Together with severe growth defects that result from *DPH5* overexpression toxicity in either *dph7* cells or in mutants affecting the first step of the pathway (Figure 1B), the study provides strong evidence that enhanced binding of unmodified or incompletely modified EF2 due to higher-than-normal Dph5 levels is inhibitory to the essential function of the translation factor (Figure 1B). Intriguingly, enhanced occupancy of EF2 by Dph5 in the absence of Dph7 appears to stabilize the diphthine modification, which in *dph6 *mutants with normal EF2-Dph5 interaction is prone to elimination of the trimethylamino group. So, in addition to its diphthine synthase role in the second pathway step (Figure 1A), Dph5 can interfere with cell growth when the stepwise generation of diphthamide on EF2 is absent or incomplete. Based on these findings, the study by Uthman *et al.* proposes an additional regulatory role for Dph5, which involves binding to newly synthesised EF2 in order to exclude it from functioning in translation until the diphthine amidation step takes place. For future validation, it will be crucial to test whether excess Dph5 can also inhibit the growth of EF2 H_699_ substitution mutants, which are unable to be diphthamide modified, in a fashion similar to its effect on *dph *mutants that block the first step of the pathway.

Physiologically, the function of diphthamide has been enigmatic. Yeast mutants unable to synthesize diphthamide are viable (Figure 1B), although some substitutions of the critical histidine in EF2 that cannot be diphthamide modified confer growth defects. However, loss of diphthamide synthesis in homozygous knockout mice lacking *DPH3/KTI11* gene function leads to embryonic lethality. Together with the association of mammalian *DPH1/OVCA1 *with ovarian cancer and neuronal development, this indicates that diphthamide modification plays an important biological role in higher eukaryal cells. In support of a role for diphthamide in EF2 function during translation, the report by Uthman *et al.* shows that incomplete diphthamide synthesis lowers the translation accuracy of EF2, causes elevated frequency of ribosomal frameshifting and affects growth in the presence of translational indicator drugs. Completion of diphthamide synthesis thus appears to assist EF2 in reading frame maintenance during translation and this role may have particular importance in multicellular organisms.

In sum, the work by Uthman *et al.* clearly has paved the way for further studies on the mechanism of diphthamide modification and its significance for EF2 functionality. In the future, it will be crucial to explore the potential role of diphthine synthase (Dph5) as a regulator of the entire pathway that is strongly suggested by the report of Uthman *et al.* As diphthamide biosynthesis is conserved from yeast to humans and genes in this pathway have been implicated in neurodegeneration and cancer, the work has ramifications for human biology in both health and disease and is of general interest in the area of posttranslational protein modification. Finally, the report presents an excellent illustration of how the latest genome-wide approaches in genetically tractable models such as *S. cerevisiae* can be exploited as a starting point for new gene discovery and the elucidation of conserved cellular processes.

